# CDK-1 and Two B-Type Cyclins Promote PAR-6 Stabilization during Polarization of the Early *C. elegans* Embryo

**DOI:** 10.1371/journal.pone.0117656

**Published:** 2015-02-06

**Authors:** Alexia Rabilotta, Marianne Desrosiers, Jean-Claude Labbé

**Affiliations:** 1 Institute of Research in Immunology and Cancer (IRIC), Université de Montréal, Montréal, Quebec, Canada; 2 Department of Pathology and Cell Biology, Université de Montréal, Montréal, Quebec, Canada; Institute of Biosciences and Technology, Texas A&amp;M Health Sciences Center, UNITED STATES

## Abstract

In the *C. elegans* embryo, formation of an antero-posterior axis of polarity relies on signaling by the conserved PAR proteins, which localize asymmetrically in two mutually exclusive groups at the embryonic cortex. Depletion of any PAR protein causes a loss of polarity and embryonic lethality. A genome-wide RNAi screen previously identified two B-type cyclins, *cyb-2.1* and *cyb-2.2*, as suppressors of *par-2(it5ts)* lethality. We found that the loss of *cyb-2.1* or *cyb-2.2* suppressed the lethality and polarity defects of *par-2(it5ts)* mutants and that these cyclins act in cell polarity with their cyclin-dependent kinase partner, CDK-1. Interestingly, *cyb-2.1*; *cyb-2.2* double mutants did not show defects in cell cycle progression or timing of polarity establishment, suggesting that they regulate polarity independently of their typical role in cell cycle progression. Loss of both cyclin genes or of *cdk-1* resulted in a decrease in PAR-6 levels in the embryo. Furthermore, the activity of the cullin CUL-2 was required to achieve suppression of *par-2* lethality when both cyclins were absent. Our results support a model in which CYB-2.1/2/CDK-1 antagonize CUL-2 activity to promote stabilization of PAR-6 levels during polarization of the early *C. elegans* embryo. They also suggest that CYB-2.1 and CYB-2.2 contribute to the coupling of cell cycle progression and asymmetric segregation of cell fate determinants.

## Introduction

**Fig 1:**
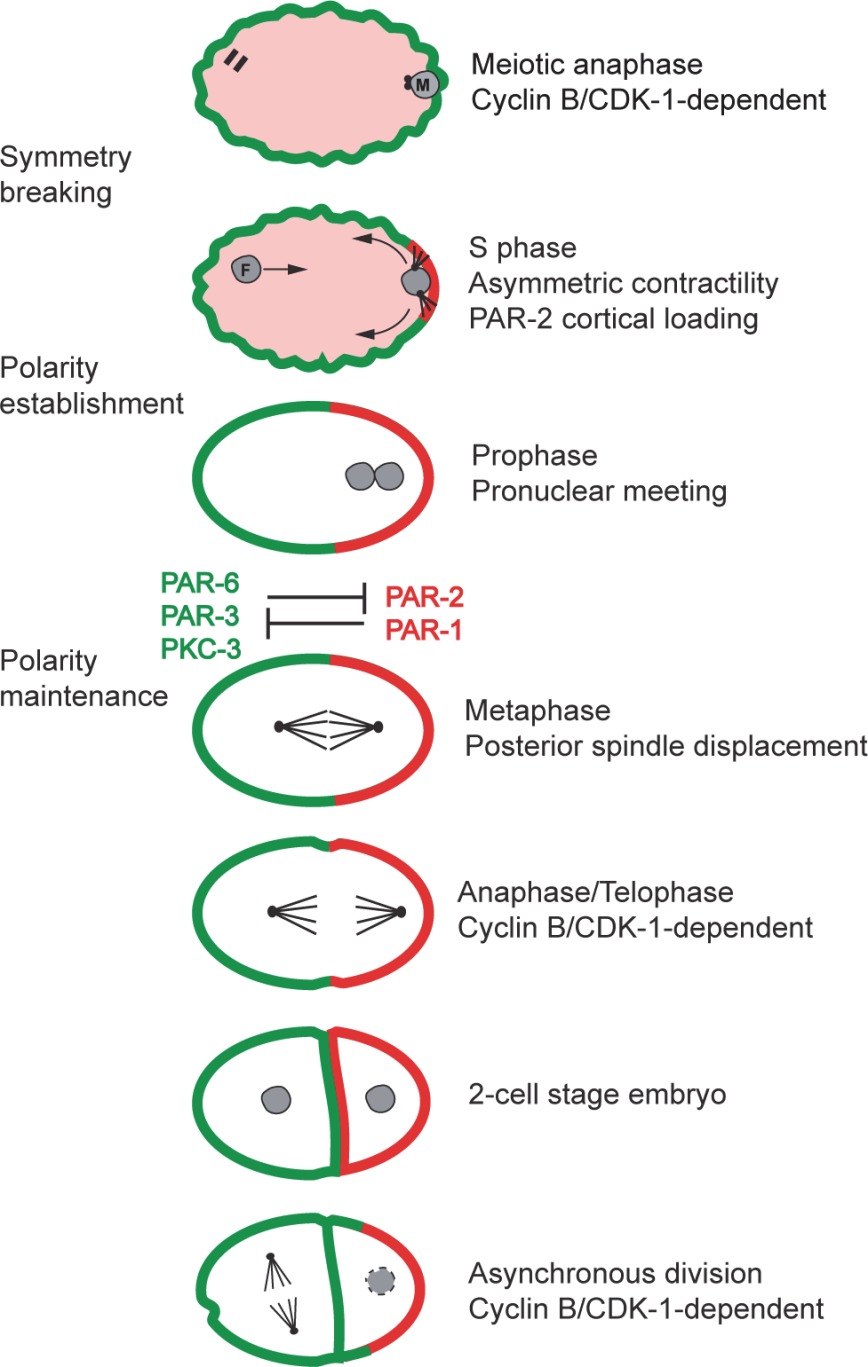
Schematic representation of PAR protein-dependent polarity establishment and maintenance during *C*. *elegans* early embryogenesis. The distribution of anterior (PAR-3, PAR-6, PKC-3, in red) and posterior (PAR-1, PAR-2, in green) PAR proteins is depicted during the first two divisions of embryonic blastomeres. In all images, anterior is to the left. M = male pronucleus; F = female pronucleus. See main text for details.

**Fig 2:**
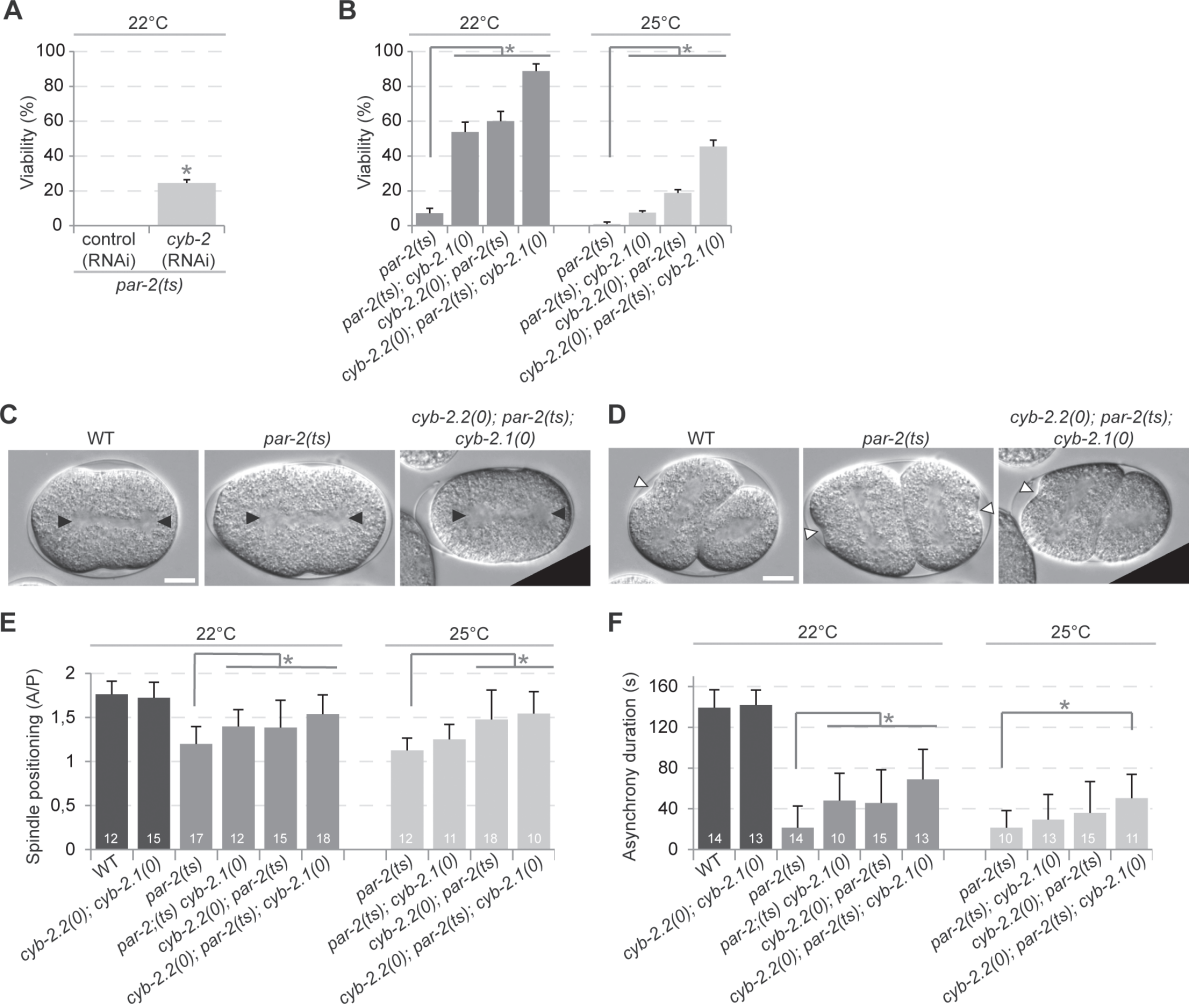
Depletion of *cyb-2*.*1* and *cyb-2*.*2* suppresses the lethality and polarity defects associated with the loss of *par-2*. (A-B) Graphs reporting the viability of embryos of the specified genotypes after RNAi (A) or genetic (B) inactivation of *cyb-2*.*1* and *cyb-2*.*2*. The values correspond to the mean percentage of hatching embryos over the total number of embryos ± SEM over three independent assays performed at the specified temperature. (C-D) DIC images from time-lapse movies of embryos grown at 22°C undergoing first (C) or second (D) division. Black arrowheads indicate centrosome positions and white arrowheads point to sites of membrane ingression during cytokinesis. In all panels, anterior is to the left. Scale bar is 10μm. (E-F) Graphs reporting the measurements for spindle position (E) and asynchrony duration (F) in embryos of the specified genotypes grown at the specified temperatures. Spindle position is expressed as a ratio of the distance between the anterior centrosome and the anterior cortex over the distance between the posterior centrosome and the posterior cortex (A/P ratio). Asynchrony duration corresponds to the time difference between the appearance of membrane ingression in AB and in P_1_. Error bars represent standard deviation over the specified number of events (n). In all panels, asterisks indicate statistical significance with control animals (p≤0.05, Student’s t-test).

Asymmetric cell division allows the unequal partitioning of cell fate determinants and is thus an essential process during development of all metazoa. The *C*. *elegans* embryo is a well-suited model to study the regulation of asymmetric division. The first division of the P_0_ zygote is asymmetric and results in two daughter cells that differ in size and fate: the larger, anterior AB blastomere, precursor of most of the ectoderm, and the smaller, posterior P_1_ blastomere that will give rise to all of the endoderm and the germ line. The second division of the embryo is also asymmetric and asynchronous between AB and P_1_, with AB entering mitosis before P_1_. This asymmetry requires the establishment and maintenance of an antero-posterior axis of polarity through asymmetric distribution of the conserved PAR polarity proteins (Fig. 1; [1,2]). While PAR-2 contains a RING finger domain typically found in E3 ubiquitin ligases, PAR-3 and PAR-6 are PDZ domain-containing proteins that can form a complex with the atypical protein kinase PKC-3 [3–7]. Establishment of embryonic polarity is triggered after the completion of maternal meiosis. It occurs via a polarized cortical flow of acto-myosin that moves cortical PAR-3, PAR-6 and PKC-3 proteins to the anterior cortex, and a concomitant microtubule-dependent deposition of PAR-2 to the posterior cortex, which results in the asymmetric enrichment of the protein kinase PAR-1 at the posterior pole [8–10]. Maintenance of respective PAR protein cortical domains relies on their mutual cortical exclusion, a process that is thought to depend on the kinase activity of the PAR-1 and PKC-3 proteins [9,11] and on proper stoichiometry between anterior and posterior PAR proteins [12,13]. Proper localization of PAR proteins in the zygote is essential for the appropriate partitioning of all anterior and posterior cell fate determinants into the two daughter cells. Accordingly, depletion of any PAR protein leads to a loss of polarity, abnormal symmetric cell divisions and embryonic lethality [1].

In embryos produced by *par-2* mutant animals (hereafter referred to as *par-2* mutant embryos), anterior PAR proteins are inappropriately localized at the posterior cortex, posterior displacement of the mitotic spindle at metaphase/anaphase is impaired and the embryo divides into two cells that are equal in size and fate and that will enter their second mitosis simultaneously [12,14]. A genome-wide RNAi screen for suppressors of *par-2(it5ts)* lethality previously uncovered genes that are or could be regulators of the anterior PAR complex [15–17]. Two of these suppressors are *cyb-2*.*1* and *cyb-2*.*2* (together referred to as *cyb-2*.*1/2*). *cyb-2*.*1/2* encode nearly identical *C*. *elegans* homologs of B-type cyclins that, along with CYB-1 and CYB-3, control meiotic and mitotic progression in the embryo [18]. In other organisms, B-type cyclins regulate cell cycle progression by interacting with a cyclin-dependent kinase (Cdk) partner, thus providing substrate specificity to the cyclin/Cdk complex. The assembly/disassembly of a functional cyclin B/Cdk1 complex during cell cycle is regulated in part by cyclin B protein levels, which rise during the G2 and M phases and drop at the metaphase-to-anaphase transition [19]. A previous analysis of *C*. *elegans* B-type cyclins function revealed that CYB-1 and CYB-3 are necessary for proper meiotic and mitotic progression in the early embryo, but that the phenocopy of CDK-1 loss of function can only be achieved after depletion of all four B-type cyclins [18]. In this study, no apparent cell cycle defect was observed after depletion of CYB-2.1/2, suggesting that CYB-1 and CYB-3 are the major regulators of cell cycle progression and can compensate for the loss of CYB-2.1/2 in this process. Interestingly, previous studies have suggested a role for key cell cycle regulators such as PLK-1, SPAT-1 and CDC-25 in asymmetric division of the *C*. *elegans* embryo [20–22]. However, although cell cycle defects were previously coupled to a loss of normal polarity establishment in the early embryo [23–25], it remains unclear whether cell cycle regulators directly control cell polarity and asymmetric cell division.

In this study, we found that loss of *cyb-2*.*1* and *cyb-2*.*2*, either singly or together, suppresses the lethality and polarity defects of *par-2(it5ts)* thermosensitive mutants without affecting the timing of *C*. *elegans* embryonic divisions, uncovering a new and specific role for these B type cyclins in cell polarity. Using molecular and genetic approaches, we demonstrate that CYB-2.1 and CYB-2.2 antagonize CUL-2 to regulate PAR-6 levels and cell polarity in parallel to NOS-3. Our results also suggest that these two B-type cyclins act in polarity with their associated kinase, CDK-1. Our results support a mechanism in which cell cycle and cell polarity are distinctly regulated but temporally coupled, by regulators such as CYB-2.1 and CYB-2.2, to ensure the accurate spatiotemporal segregation of cell fate determinants before asymmetric division.

## Materials and Methods

### Strains and alleles

Strains were maintained as described by Brenner [26] and grown at 15°C unless otherwise stated. The wild-type strain was the Bristol N2 strain. The alleles used in this study were LGI: *cyb-2*.*2(tm1969)*; LGII: *nos-3(q650)*; LGIII: *cdk-1(ne2257ts)*, *par-2(it5ts)*, *cul-2(or209ts)*, *unc-119(ed3)*; LGIV: *cyb-2*.*1(tm2027)*; LG?: *xnIs3[Ppar-6*::*par-6*::*gfp; unc-119(+)]*, *ltIs37[Ppie-1*::*mCherry*::*his-58; unc-119(+)]*, *zuIs45[Pnmy-2*::*nmy-2*::*gfp; unc-119(+)])*.

The *cyb-2*.*1(tm1969)* and *cyb-2*.*2(tm2027)* alleles respectively have a 638bp and 424bp deletion in the promoter and 5’ region of each gene. Quantitative RT-PCR analysis of transcript levels from each locus revealed that they are found in trace levels in mutant animals (S1 Fig. and see below), supporting the notion that both alleles are null. Double, triple and quadruple mutants were generated by genetic crosses and genotypes were assessed either visually by phenotypic scoring or molecularly by PCR, using pairs of primers specific to each locus.

### Quantitative RT-PCR analysis

Young wild-type adults or *cyb-2*.*1/2* mutant adults were rinsed in M9 buffer (22 mM KH_2_PO_4_, 42 mM Na_2_HPO_4_, 85 mM NaCl, 1 mM MgSO_4_) and lysed in Trisol (Gibco) by repeated thawing followed by Trisol-chloroform extraction. RNA was purified from these extracts using the RNeasy Mini kit (Qiagen) according to the manufacturer’s recommendation. The quantity and purity of RNA samples was validated using ThermoFisher’s NanoDrop and Agilent's Bioanalyzer instruments (Nano Chip). After DNAse treatment, first strand cDNA was synthesized with random primers using the High Capacity cDNA Reverse Transcription Kit (Life Technologies). Quantitative reverse transcription-polymerase chain reaction (qRT-PCR) was then performed with the PerfeCTa SYBR Green SuperMix (Quanta BioSciences) using a Viia7 real-time cycler instrument (Life Technologies). Melting curve analysis was performed after the final cycle to examine the specificity of each reaction and a no-template control was done for every primer pair. Two primer pairs were designed to amplify common regions in *cyb-2*.*1* and *cyb-2*.*2* (pair 1: TTGTCTCCAAGGCGGAAT + GGCAAGTAAATATCTTCGAATTTGG, and pair 2: GATTCGGTCATGTCTACGGTT + GATGCAGCAGTCTTCGGATTA), and one primer pair specific for *cyb-1* (pair 3: AGATGGCTAAGCACGGAAAC + TTTCGACTGGAGCTGGATTG). Endogenous controls were performed using primer pairs specific for *cdc-42* (ctgctggacaggaagattacg + ctcggacattctcgaatgaag) and *pmp-3* (gttcccgtgttcatcactcat + acaccgtcgagaagctgtaga) as described previously [27]. For each assay, a standard curve was performed to ensure a 90–110% range in efficacy. Three independent extracts were analyzed in duplicate for each genotype. Relative gene expression for each sample was normalized to that of endogenous controls using the RQ = 2^-∆∆CT^ equation in the Expression Suite software (Life Technologies). Error bars correspond to the ratio of RQmin over RQmax (2^(RQ-SEM)^ / 2^(RQ+SEM)^) using a 95% confidence interval.

### Lethality suppression assays

Protein depletion by RNAi feeding was performed as described previously [28] using individual clones from Julie Ahringer’s library. All clones were verified by sequencing. Clone C32E12.1 was used as RNAi control since it has no effect on embryonic development and does not suppress par-2(it5) defects [16]. Suppression assays on all mutant backgrounds were performed in triplicates as described previously [15]. Briefly, 15–20 L4 animals were transferred to a plate seeded with either OP50 bacteria or HT115 bacteria expressing dsRNA, depending on the assay, and the plate was incubated at the desired temperature for 24h. Nine animals were then transferred to three plates prepared like the first one (3 worms per plate) and allowed to lay eggs for an additional 16h before they were removed. The plates were kept at the same temperature for a minimum of 24h to allow completion of embryonic development. Hatching frequency was assessed by counting the number of unhatched eggs over the total number of progeny on each plate.

### Time-lapse microscopy

The timing of early embryonic events was assessed by differential interference contrast microscopy. Embryos were mounted and time-lapse images were acquired every 5 seconds with a Zeiss Axio-Imager Z1 microscope, as described previously [29]. Spindle orientation and positioning was determined by measuring the position of the two centrosomes along the antero-posterior axis as described previously [15]. Nuclear envelope breakdown and cortical ingression at cytokinesis were scored by visual inspection.

Time-lapse imaging of fluorescent specimens was done with a CoolSnap HQ^2^ camera (Photometrics) mounted on a Nikon Swept Field Confocal microscope (Nikon Canada, Mississauga, ON, Canada; and Prairie Technologies, Madison, WI, USA) and using a 63X objective. [30]To assess the velocity of cortical NMY-2::GFP flows during the polarization phase, 16 confocal sections (each separated by 0.5 μm) were acquired at the upper cortex and the anterior displacement of NMY-2::GFP foci over time was measured by kymograph analysis as described previously [30]. Analyses were performed using Image J software (NIH).

### Immunofluorescence

For immunofluorescence analysis, embryos were fixed in methanol and stained as described previously [29]. Images were acquired using a Zeiss LSM 510 confocal microscope. The primary antibodies used were rabbit anti-PAR-6 (1/50, [15]) and mouse P4A1 anti-PAR-3 (1/150, Developmental Studies Hybridoma Bank). Secondary antibodies were Alexa488-coupled goat-anti-rabbit and Alexa546-coupled goat-anti-mouse (1/500 each, Invitrogen). The cortical distribution of PAR-3 and PAR-6 proteins was measured using Image J software by plotting fluorescence intensity values of a 10 pixel-thick line drawn around the entire cortex of the embryo. This produced a fluorescence intensity profile where a broad peak of intensity corresponding to the entire anterior cortex is bordered by regions of low/background intensity in the posterior pole. The two points on each side of the broad peak where fluorescence intensity starts to rise were considered as PAR protein cortical boundaries and were determined as the intersection between the average fluorescence intensity of the posterior cortex and that of the slopes on each side of the peak. PAR protein domain size in each embryo was expressed as the distance between the anterior pole and the average of the two intersecting points relative to embryo length.

### Western blot analyses

To assess PAR-6 levels, synchronized animals were grown at 15°C until they reached the L4 stage and were then transferred to 20°C for 24 hrs. Animals were collected in M9 buffer and embryos were recovered by sodium hypochlorite treatment. Extracts were obtained by grinding embryos in liquid nitrogen and resolved by SDS-PAGE. Western blot analysis was performed as described previously [15] on three extracts prepared independently, and three blots were made for each extract. The primary antibodies were rabbit anti-PAR-6 (1/500, [15]) and mouse anti-tubulin (DM1A, 1/5000, Sigma). Secondary antibodies were HRP-coupled goat anti-mouse and goat anti-rabbit (1/10000, Bio-Rad).

## Results

### *cyb-2*.*1* and *cyb-2*.*2* are suppressors of *par-2(it5ts)* lethality and polarity defects

Depletion of *cyb-2*.*1* or *cyb-2*.*2* by RNAi suppresses *par-2(it5ts)* embryonic lethality at the semi-restrictive temperature of 22°C (Fig. 2A; [16]). However, *cyb-2*.*1* and *cyb-2*.*2* share 97% identity in their nucleotide sequence, making them effective co-targets in RNAi assays. To determine whether *cyb-2*.*1* and *cyb-2*.*2* function redundantly in polarity, we generated double and triple mutants between *par-2(it5ts)* and null alleles for each cyclin-encoding gene (see Materials and Methods and S1 Fig.) and tested the resulting effect on embryonic viability. While 7% of embryos produced by *par-2(it5ts)* animals (hereafter referred to as *par*-2 mutant embryos) at the semi-restrictive temperature of 22°C were viable, animals double mutant for *par-2(it5ts)* and either *cyb-2*.*1(tm1969)* or *cyb-2*.*2(tm2027)* respectively produced 54% and 60% of viable progeny (Fig. 2B). Viability was significantly higher for *cyb-2*.*2(tm1969); par-2(it5ts)*; *cyb-2*.*1(tm2027)* triple mutant embryos with 88% hatching frequency, revealing an additive effect on *par-2(it5ts)* suppression when both cyclins are removed (Fig. 2B). Similar results were obtained when animals were grown at the fully restrictive temperature of 25°C, where the 45% hatching frequency of triple mutant embryos was statistically additive when compared to either double mutant condition (Fig. 2B).

*par-2* mutant embryos show cellular defects consistent with a loss of polarity such as central mitotic spindle positioning at the first embryonic division and loss of cell cycle asynchrony between AB and P_1_ divisions [12,14]. These polarity defects were partially suppressed in double and triple mutants for *par-2*, *cyb-2*.*1* and/or *cyb-2*.*2* at the semi-restrictive temperature of 22°C, and in the triple mutant at the restrictive temperature of 25°C (Fig. 2C-F). Together, these results indicate that both CYB-2.1 and CYB-2.2 function in embryonic polarity during asymmetric division of *C*. *elegans par-2* mutant embryos.

### CYB-2.1 and CYB-2.2 function with CDK-1 in embryonic polarity

**Fig 3:**
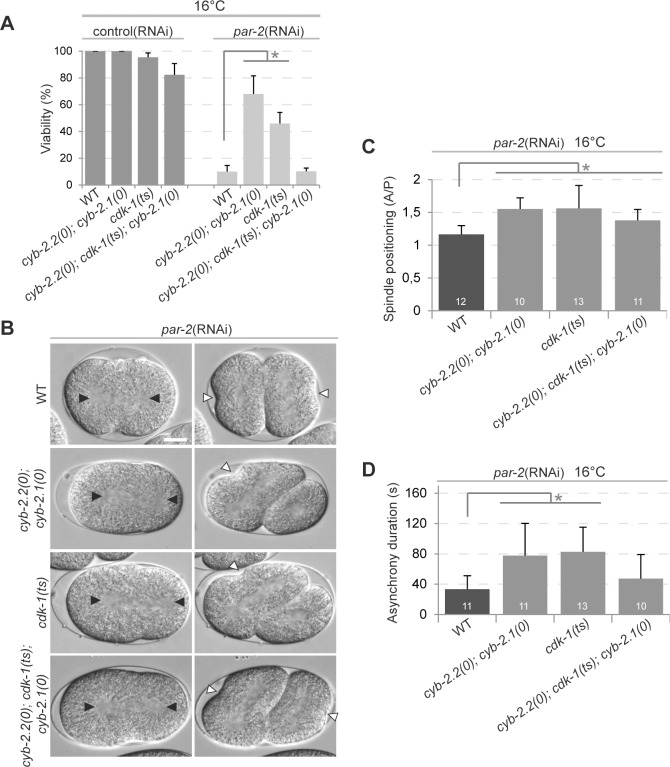
Mutation in the cyclin B-associated kinase CDK-1 suppresses *par-2*(RNAi) lethality and polarity defects. (A) Graph reporting the viability of embryos of the specified genotypes after control(RNAi) or *par-2*(RNAi) treatment. The values correspond to the mean percentage of hatching embryos over the total number of embryos ± SEM over three independent assays performed at 16°C. (B) DIC images from time-lapse movies of *par-2*(RNAi)-depleted embryos grown at 16°C undergoing first (left) and second (right) division. In all panels, anterior is to the left. Scale bar is 10μm. Black arrowheads indicate centrosome positions and white arrowheads point to sites of membrane ingression during cytokinesis. (C-D) Graphs reporting the measurements for spindle position (C) and asynchrony duration (D) in embryos of the specified genotypes grown at 16°C. Error bars represent standard deviation over the specified number of events (n). In all panels, asterisks indicate statistical significance with control animals (p≤0.05, Student’s t-test).

Cyclins typically regulate cell cycle progression by interacting with a specific Cdk partner, providing substrate specificity for the phosphorylation of distinct targets. To ask if CYB-2.1 and CYB-2.2 function with CDK-1 to regulate embryonic polarity, we used animals mutant for the thermosensitive *cdk-1(ne2257)* allele to determine if CDK-1 has a role in embryonic polarity. The *ne2257* allele was previously shown to code for a protein bearing an isoleucine/phenylalanine substitution at residue 173, within the T loop of the kinase domain, that retains normal kinase activity *in vitro* [31]. Embryos mutant for *cdk-1(ne2257)* failed to hatch when grown at restrictive temperature, however they were reported to achieve normal cell divisions during development [31]. As the penetrance of lethality and polarity defects was similar between *par-2*(RNAi) and *par-2*(it5ts) mutant animals raised at restrictive temperature, we used RNAi to deplete PAR-2 from *cdk-1(ne2257)* embryos, to avoid the complications of working with two temperature-sensitive alleles. We found that at the temperature of 16°C, at which 95% of *cdk-1(ne2257)* embryos hatched, the *cdk-1(ne2257)* allele restored the viability of *par-2*(RNAi) embryos and suppressed *par-2*(RNAi)-related polarity defects in a manner similar to *cyb-2*.*2; cyb-2*.*1* double mutants (Fig. 3). These results support the notion that CYB-2.1 and CYB-2.2 regulate polarity through their activity as part of a cyclin/Cdk complex.

We next asked if the suppression of *par-2*(RNAi) phenotypes in *cyb-2*.*2; cdk-1*; *cyb-2*.*1* triple mutants was additive or equal to that in *cyb-2*.*1/2* and *cdk-1* mutants but found that their combined effect was not additive for suppression of lethality and loss of asynchrony in *par-2*(RNAi) embryos. Rather, we observed a slight increase in embryonic lethality for the triple mutant treated with control(RNAi) and no suppression when treated with *par-2*(RNAi) (Fig. 3). This suggests that there is a threshold of cyclin/Cdk1 activity below which embryonic development is perturbed, and that suppression of *par-2*(RNAi) requires a level of CDK-1 activity higher than that needed to allow most embryos to complete embryogenesis.

### CYB-2.1 and CYB-2.2 regulate anterior PAR protein localization

**Fig 4:**
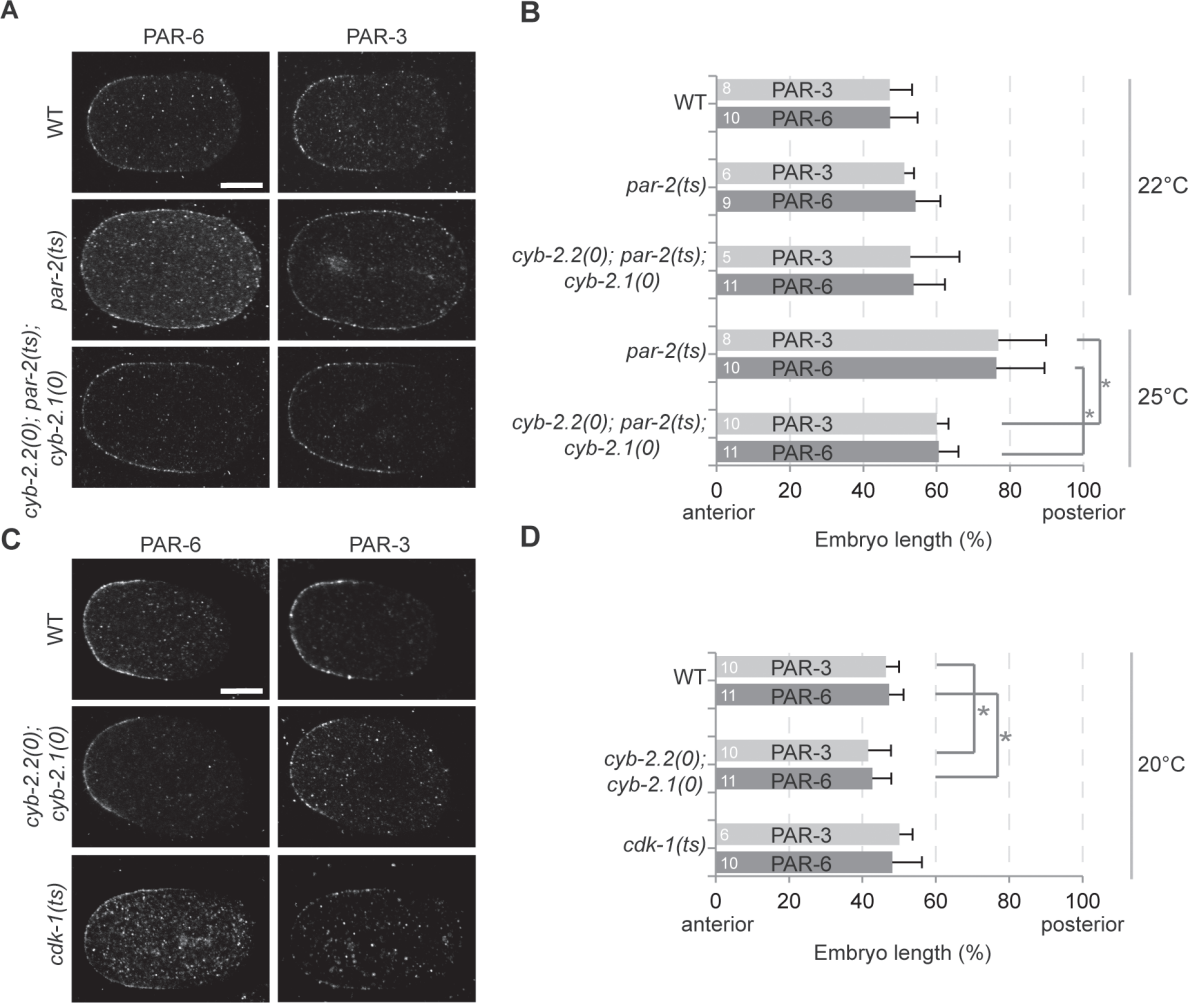
CYB-2.1/2 affect anterior PAR protein localization in the 1-cell embryo. (A, C) Images of embryos of the specified genotypes grown at 25°C (A) or 20°C (C), fixed during mitosis and stained with anti-PAR-6 (left panels) or anti-PAR-3 (right panels) antibodies. In all panels, anterior is to the left. Scale bar is 10μm. (B, D) Graphs reporting the cortical distribution (in % embryo length) of endogenous PAR-6 and PAR-3 along the antero-posterior axis in embryos of the specified genotypes grown at 22°C, 25°C (B) or 20°C (D) and fixed during mitosis. Error bars represent standard deviation over the specified number of events (n). In all panels, asterisks indicate statistical significance with control animals (p≤0.05, Student’s t-test).

Since *par-2* depletion results in a loss of PAR protein asymmetry, we next tested the effect of CYB-2.1/2 on PAR protein localization in the early embryo. In wild-type embryos undergoing mitosis, anterior PAR proteins are restricted to the anterior cortex, occupying ∼50% of embryo length (Fig. 4A-B). In *par-2(it5ts)* mutants grown at restrictive temperature, PAR-3 and PAR-6 localization is displaced towards the posterior cortical pole, each occupying ∼75% of embryo length (Fig. 4B). Mutating *cyb-2*.*1* and *cyb-2*.*2* suppressed this defect and restored PAR-3 and PAR-6 cortical distribution to 61% and 60% of embryo length, respectively (Fig. 4B). While, *cyb-2*.*2*; *cyb-2*.*1* double mutant embryos themselves displayed a small but statistically significant anteriorization of cortical PAR-3 and PAR-6 proteins at prophase compared to wild-type embryos (Fig. 4C-D), *cdk-1* mutant embryos did not show this phenotype (Fig. 4D). These results indicate that the effect of *cyb-2*.*1/2* loss-of-function on PAR protein localization is modest, but effectively manifests itself when polarity is compromised.

### CYB-2.1 and CYB-2.2 regulate polarity independently of cell cycle progression

Embryonic polarization initiates after the meiosis-to-mitosis transition, a cell cycle-dependent event governed by B-type cyclins and CDK-1 (Fig. 1; [23,25,32,33]). As cell cycle perturbations were previously reported to result in polarity defects [24], this raised the possibility that CYB-2.1/2 and CDK-1 could regulate polarity through their activity in controlling cell cycle progression. To address this, we monitored the timing and progression of several cellular events in embryos mutant for *cyb-2*.*1/2* or *cdk-1*. We found that the initiation, duration and velocity of cortical acto-myosin flows were unchanged in *cyb-2*.*1/2* mutants compared to control, indicating that CYB-2.1/2 do not impinge on cytoskeleton-dependent polarizing events or on the timing of polarity initiation following meiosis II anaphase (S2 Fig.). We also found that the duration of mitotic progression during the first two divisions of *cyb-2*.*1/2* mutant embryos was equal to that of wild-type embryos (S3A Fig.), and there was no cumulative cell cycle progression defect in *cyb-2*.*1/2* mutants embryos, as the initiation of epidermal enclosure in wild-type and *cyb-2*.*1/2* mutants occurred in both cases ∼5 hours after the second embryonic division (as scored in 8 embryos each, data not shown). We conclude that mutating *cyb-2*.*1* and *cyb-2*.*2* does not impair cell division timing and can suppress the defects of *par-2* mutants without perturbing cell cycle progression.

Analysis of *cdk-1* mutants grown at 16°C revealed mild defects in cell cycle progression and in spindle positioning during the first embryonic division (S3A–B Fig.). However, other polarity-related phenotypes, such as the cell cycle asynchrony between AB and P_1_ divisions and the progression of pronuclear migration, were normal in these embryos indicating, as reported previously [31], that polarity is not overly compromised (S3C Fig.). Similar defects for *cdk-1* mutant embryos were observed at 20°C, at which 67% of *cdk-1(ne2257)* embryos hatched, although cell cycle asynchrony at the 2-cell stage was significantly decreased as compared to wild-type embryos grown at this temperature (S3 Fig.). These results indicate that, contrary to disruption of *cyb-2*.*1/2*, the *cdk-1(ne2257)* allele results in mild but significant defects in cell cycle progression.

All four *C*. *elegans* B-type cyclins were previously shown to function semi-redundantly in the regulation of cell cycle progression [18]. We therefore asked whether depleting the two other cyclins, CYB-1 and CYB-3, could suppress the lethality of *par-2* mutants. We found that depletion of CYB-1 by RNAi caused perturbations in cell cycle progression and resulted in embryonic lethality in wild-type embryos, but did not restore proper spindle orientation and positioning in *par-2* mutants (S4A–B Fig.). The increase in cell cycle asynchrony observed in CYB-1-depleted *par-2* mutants could be a consequence of CYB-1 depletion itself, as it has a similar effect on cell cycle asynchrony in wild-type embryos. Depleting CYB-3 did not suppress the embryonic lethality of *par-2* mutants (data not shown). These results indicate that perturbing cell cycle progression by depleting other cyclins is not sufficient to suppress the phenotypes of *par-2* mutants. As *cyb-2*.*1/2* double mutants display suppression of *par-2* phenotypes but have normal cell cycle progression, these results also indicate that CYB-1 and/or CYB-3 can compensate for the cell cycle activity of CYB-2.1/2 but not for their role in cell polarity.

### CYB-2 and CDK-1 regulate PAR-6 levels through the Cullin CUL-2, but independently of NOS-3

**Fig 5:**
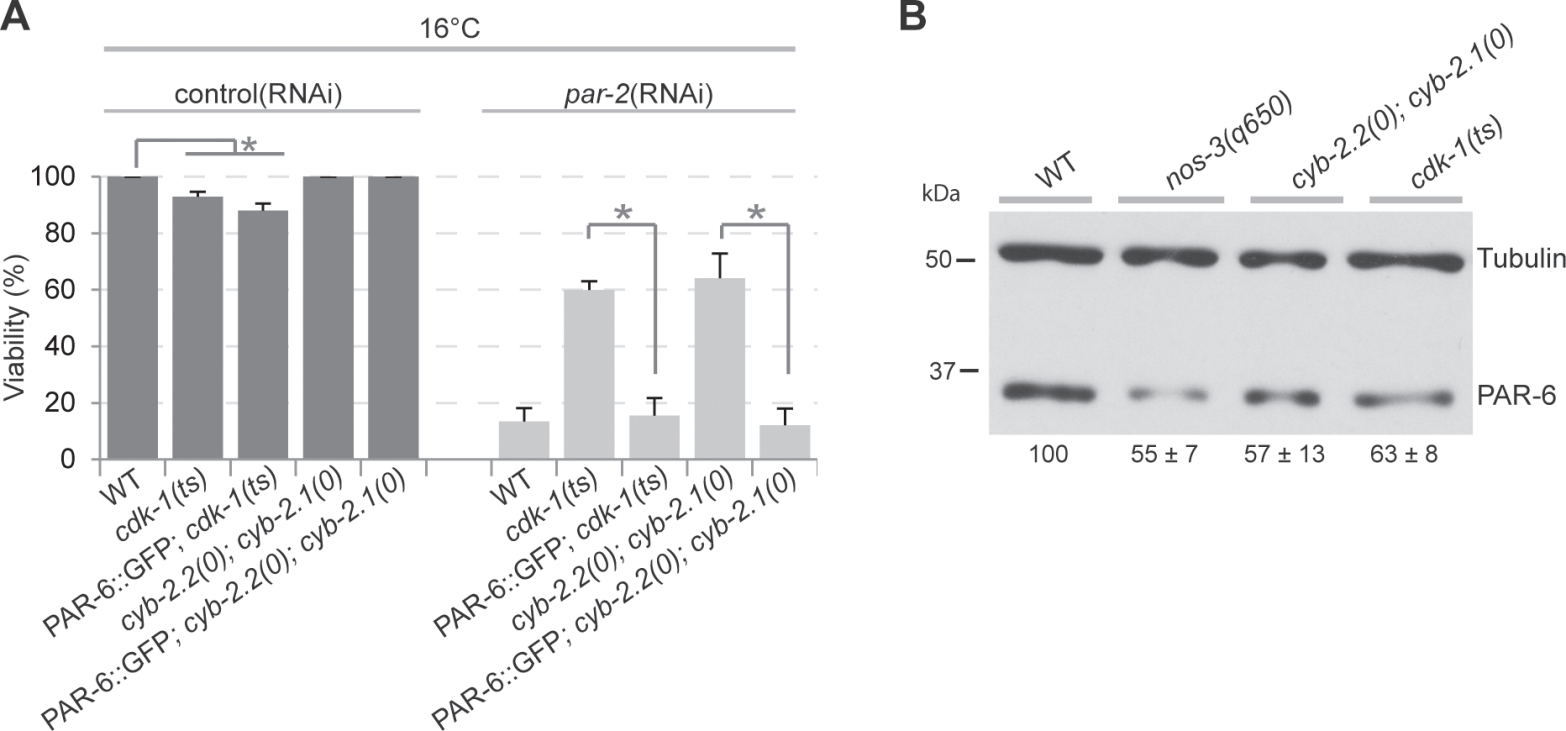
CYB-2.1/2 and CDK-1 regulate PAR-6 levels in the early embryo. (A) Graph reporting the viability of embryos of the specified genotypes after control(RNAi) or *par-2*(RNAi) treatment. The values correspond to the mean percentage of hatching embryos over the total number of embryos ± SEM over three independent assays performed at 16°C. (B) Western blot analysis of extracts from embryos of the specified genotypes grown at 20°C and revealed with anti-PAR-6 and anti-alpha-tubulin antibodies. The value under each lane corresponds to the ratio of PAR-6 over alpha-tubulin intensity ± SEM in three independent experiments, with the mean value normalized to WT levels.

Previous results have revealed that regulation of PAR protein levels is an effective mean to regulate PAR protein distribution and cell polarity [13,16,34]. Indeed, PAR-6 levels are actively regulated *in vivo* by a Cullin-based E3 ubiquitin ligase pathway that can modulate PAR protein distribution [17]. As PAR-3 and PAR-6 distribution is affected in *cyb-2*.*1; cyb-2*.*2* double mutants, we asked whether PAR-6 levels are reduced in these animals. We first determined whether suppression of *par-2*(RNAi) lethality by mutations in *cyb-2*.*1/2* or *cdk-1* was affected in animals that overexpress PAR-6::GFP, as done previously [17]. We found that while the embryonic lethality caused by PAR-2 depletion was strongly reduced in animals mutant for *cyb-2*.*1/2* or *cdk-1*, it was not reduced in the same mutants overexpressing PAR-6::GFP (Fig. 5A). Furthermore, western blot analyses of embryonic extracts revealed that PAR-6 protein levels were significantly decreased in *cyb-2*.*1/2* and *cdk-1(ne2257)* mutants, to 57% and 63% of wild-type levels, respectively (Fig. 5B and S5 Fig.). These levels are comparable to what was observed in embryos mutant for *nos-3*, a known suppressor of *par-2* lethality and regulator of PAR-6 levels in *C*. *elegans* (Fig. 5B; [16,17]). These results indicate that CYB-2.1/2 and CDK-1 are required to maintain high levels of PAR-6 in the early embryo.

**Fig 6:**
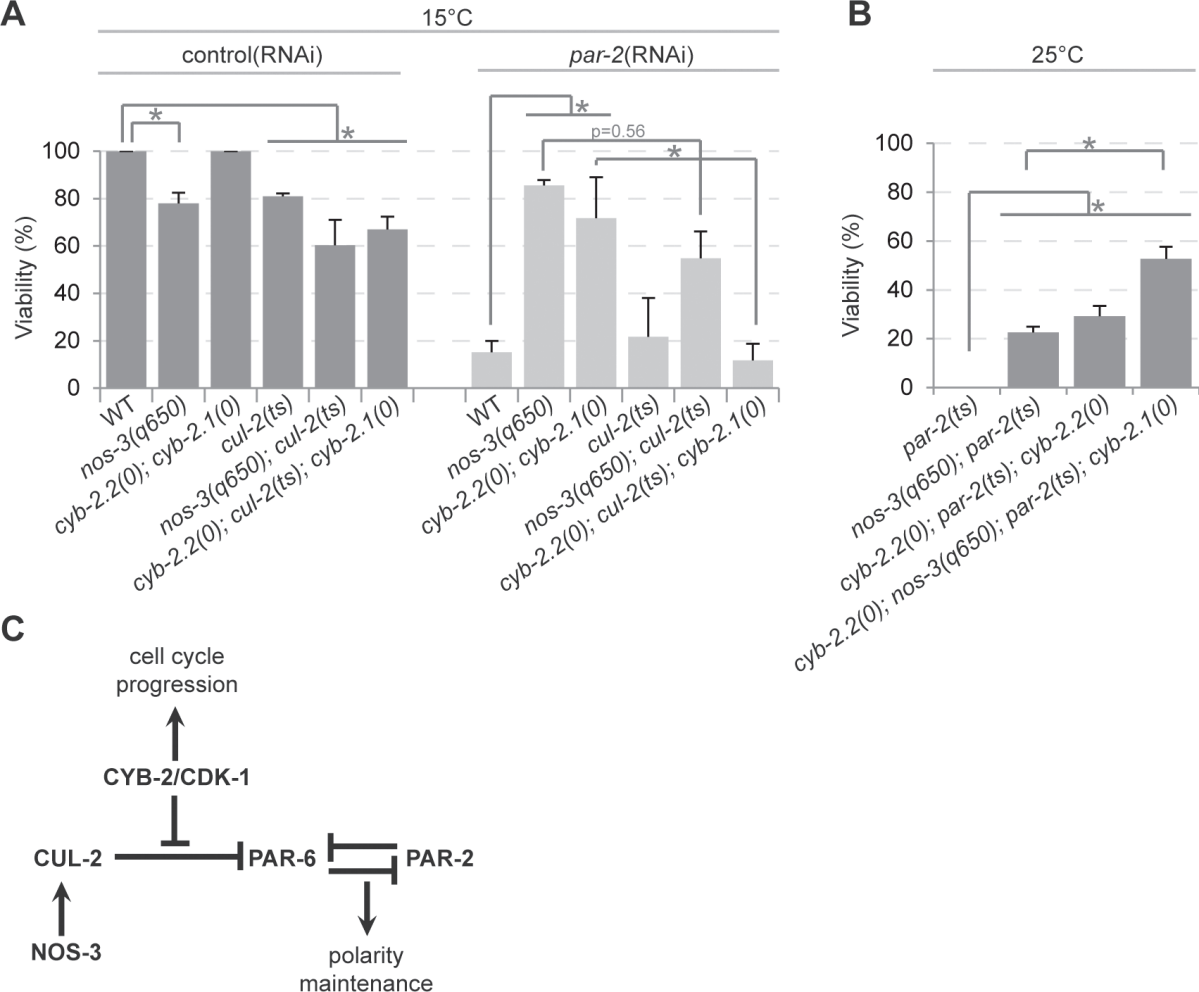
CYB-2.1/2 act with the Cullin CUL-2 but independently of NOS-3 in the embryonic polarity pathway. (A-B) Graph reporting the viability of embryos of the specified genotypes after control(RNAi) or *par-2*(RNAi) treatment. The values correspond to the mean percentage of hatching embryos over the total number of embryos ± SEM over three independent assays performed at 15°C (A) or 25°C (B). In all panels, asterisks indicate statistical significance with control animals (p≤0.05, Student’s t-test) (C) Model depicting the proposed dual role of CYB-2.1/2/CDK-1 in the regulation of cell polarity and cell cycle progression during asymmetric division of the *C*. *elegans* embryo (see discussion for details).

The levels of PAR-6 protein were previously proposed to depend on proteasome-mediated degradation following ubiquitination by an E3 ubiquitin ligase complex composed of the Cullin subunit CUL-2, the co-activators FEM-2 and FEM-2 and the substrate recognition subunit FEM-1 [17,35]. To determine if CYB-2.1/2 regulate PAR-6 levels through the CUL-2 pathway, we asked if *cyb-2*.*1/2*-dependent suppression of *par-2*(RNAi) lethality depends on the presence of CUL-2. CUL-2 activity was modulated by using animals mutant for the thermosensitive *cul-2(or209)* allele, which produce 80% of viable progeny at permissive temperature (Fig. 6A; [36]). While embryonic viability was 72% in *cyb-2*.*1; cyb-2*.*2* double mutants depleted in PAR-2, we observed 12% embryonic viability in *cyb-2*.*2; cul-2; cyb-2*.*1* triple mutants depleted of PAR-2. This severe decrease in embryonic viability is significantly higher than that observed in *cyb-2*.*2; cul-2; cyb-2*.*1* triple mutant animals treated with control(RNAi) (67%; Fig. 6A). We conclude that CUL-2 activity is necessary for suppression of *par-2* embryonic lethality by *cyb-2*.*1/2* and that CYB-2.1/2 may regulate PAR-6 levels through CUL-2.

NOS-3 was previously shown to regulate PAR-6 levels through CUL-2 activity, perhaps by regulating the translation of the FEM-3 co-activator [17]. We therefore used genetic analysis to determine if CYB-2.1/2 function through NOS-3 in the polarity pathway. While *par-2(it5ts)* animals produced no viable progeny at restrictive temperature, embryonic viability in *nos-3; par-2* double mutants and *cyb-2*.*2; par-2; cyb-2*.*1* triple mutants was 23% and 29%, respectively. Embryonic viability in *cyb-2*.*2; nos-3; par-2; cyb-2*.*1* quadruple mutants increased to 53%, a value statistically additive between that of the double and triple mutant animals (Fig. 6B). Based on the additive nature of this interaction, we conclude that CYB-2.1/2 act with CUL-2 but independently of NOS-3 to regulate PAR protein-dependent polarity.

## Discussion

In this study, we reported that two homologs of B-type cyclins, CYB-2.1 and CYB-2.2, and their associated kinase CDK-1 have a role in PAR protein-dependent cell polarity. Mutations in *cyb-2*.*1*/*2* and *cdk-1* suppressed the lethality of *par-2* mutants and decreased PAR-6 protein levels in the embryo. Epistatic analysis revealed that the role of *cyb-2*.*1/2* in polarity depends on *cul-2*, suggesting that CYB-2.1 and CYB-2.2 regulate PAR-6 degradation, likely through a CUL-2 based E3 ubiquitin ligase complex. A previous study has shown that NOS-3, another suppressor of *par-2*, also controls the levels of PAR-6 in the embryo through a CUL-2-based ubiquitin complex that uses FEM-2 and FEM-3 as co-activators and FEM-1 as substrate-specific adaptor [17]. Interestingly, while our results are consistent with this model, we found that CYB-2.1/2 regulate PAR-6 levels independently of NOS-3. Together with other studies on regulators of PAR-6 levels [13,15,17,37], our work highlights a network of molecular and genetic interactions that tightly controls PAR-6 levels during polarization of the early *C*. *elegans* embryo (Fig. 6C).

Our results favor a model in which NOS-3 and CYB-2.1/2 have different targets in the regulation of PAR-6 degradation. NOS-3 is a known translational repressor of FEM-3, suggesting that it regulates the activity of the Cullin complex by regulating FEM-3 levels [17,38]. In this study, we did not find the direct target of the CYB-2.1/2/CDK-1 complex in polarity. As a master post-translational regulator during mitosis, CDK-1 could possibly regulate PAR-6 levels directly or indirectly through phosphorylation. Mass spectrometry analysis of PAR-6 immunoprecipitated from embryonic extracts revealed two phosphorylation sites and one potential ubiquitination site on the protein. However, mutating each of these sites did not perturb PAR-6 localization in the embryo and transgenic expression of each mutant proteins was sufficient to rescue *par-6(RNAi)* embryonic lethality (data not shown). While the target(s) of CDK-1 in the *C*. *elegans* polarity pathway remains unknown, the role of this master kinase in the regulation of polarity protein stability was previously observed in other cell types. For example, phosphorylation of the human Discs large homologue 1 (DLG1) protein by CDK1 was shown to affect its susceptibility to ubiquitination [39].

CDK-1 was previously implicated in the degradation of OMA-1, an embryonic cell fate determinant whose proteolysis occurs after completion of the first division and, like PAR-6, was proposed to require CUL-2 activity [31,40]. CDK-1 thus functions as a negative regulator for PAR-6 degradation and a positive regulator for OMA-1 proteolysis. One difference that may explain this distinct outcome for each protein could be linked to the cyclin partner with which CDK-1 impinges on each process, as PAR-6 stability depends on CYB-2.1/2 whereas OMA-1 responds to CYB-3 [31]. The CUL-2 complexes regulating PAR-6 and OMA-1 stability function via distinct co-activators and substrate-specific adaptors [31,35], and CDK-1 could also act directly on the CUL-2 E3 ligase complex to modify its activity or substrate specificity through one of these adaptors, for instance by differential regulation of a subunit like FEM-1 that contains a Cdk1 consensus phosphorylation site. Understanding whether different cyclins provide substrate specificity or control the activity of the cyclin B/Cdk1 complex, and whether this directly modulates the activity of the CUL-2 complex during embryonic polarization will require further investigation.

Previous studies have revealed a role in *C*. *elegans* embryonic polarity for a number of mitotic regulators including PLK-1, SPAT-1 and components of the APC complex [20–22,24]. As inactivation of these proteins greatly affects cell cycle progression, it remained unclear if the effect of these mutations on cell polarity were a direct consequence of cell cycle defects. While CYB-2.1 and CYB-2.2 were previously shown to participate in the control of cell cycle progression during *C*. *elegans* embryogenesis [18], we found that *cyb-2*.*1/2* mutant embryonic blastomeres have wild-type cell cycle timing. This indicates that the two other B-type cyclins, CYB-1 and CYB-3, can compensate for the loss of CYB-2.1/2 activity in the regulation of cell cycle progression but not cell polarization. This uncoupling could be due to a different threshold requirement in cyclin B/Cdk1 activity for these two processes. This notion is supported by our finding that *par-2*(RNAi) lethality was not suppressed in *cyb-2*.*1/2; cdk-1* triple mutants and is further compatible with findings made in Drosophila neuroblasts, where the asymmetric localization of the polarity proteins Inscuteable and Prospero was shown to depend on a threshold of cyclin B/Cdk1 activity that is higher than the one required for cell division [41]. Alternatively, the uncoupling between cell cycle progression and cell polarization observed in *cyb-2*.*1/2* mutants could point to a specific involvement of CYB-2.1/2 in cell polarity. Interestingly, while depleting CYB-1 resulted in severe cell cycle defects, consistent with its main involvement in meiotic and mitotic progression [18], it did not restore viability or polarity in *par-2* mutants, and neither did CYB-3 depletion. These results indicate that perturbing cell cycle progression does not systematically lead to the suppression of *par-2* defects, further suggesting an uncoupling between these two cellular processes.

## Supporting Information

S1 Fig Quantitative transcriptional analysis at the *cyb-2*.*1* and *cyb-2*.*2* loci in wild-type and *cyb-2*.*1/2* double mutant animals. A) Schematic organization of the *cyb-2*.*1* (top), *cyb-2*.*2* (middle) and *cyb-1* (bottom) loci and exon cDNA produced from each transcribed mRNA. Exons are depicted as light or dark grey boxes and non-coding regions (promoters, introns, flanking sequences) as lines. Position of deletions for the *cyb-2*.*1(tm2027)* and *cyb-2*.*2(tm1969)* alleles are depicted in red and the regions amplified by qPCR with each primer pair are shown in green. B) Relative gene expression ratios averaged from three biological samples for each strain. Bars indicate the 95% confidence interval of the mean (non-overlapping intervals denote significant differences at the 0.05 level). In each strain, expression levels of cyclin genes were normalized to the mean expression of *cdc-42* and *pmp-3*.
(TIF)

S2 Fig CYB-2.1/2 do not regulate the timing or velocity of acto-myosin-dependent cortical flows. (A) Fluorescence images from time-lapse movies of control embryos undergoing meiosis II anaphase (left) and polarity establishment (right) and expressing NMY-2::GFP (green) and mCherry::H2B (red). White arrowheads point to the cortical domain boundary that is devoid of NMY-2::GFP. (B-C) Graphs reporting the time between meiotic II anaphase onset and contractile polarity establishment (B) and the velocity of NMY-2::GFP foci (C) in embryos of the specified genotypes grown at 22°C. Error bars represent standard deviation over the specified number of events (n). Values were not statistically different from control animals (p>0.05, Student’s t-test).
(TIF)

S3 Fig Cell cycle durations and polarity-related phenotypes in *cyb-2*.*1/2* and *cdk-1* mutant embryos. (A-C) Graphs reporting the duration of mitosis during the first two embryonic divisions (A), the measurements in 1-cell embryos of spindle positioning (B) and duration of asynchrony (C) in animals of the specified genotypes grown at the specified temperature. Error bars represent the standard deviation over the specified number of events (n). In all panels, asterisks indicate statistical significance with control animals (p≤0.05, Student’s t-test).
(TIF)

S4 Fig Depletion of CYB-1 does not restore embryonic polarity in *par-2* mutants. (A-B) Graphs reporting the measurements of spindle positioning (A) and duration of asynchrony (B) in 1-cell embryos of the specified genotypes grown at 22°C. Error bars represent the standard deviation over the specified number of events (n). In all panels, asterisks indicate statistical significance with control animals (p≤0.05, Student’s t-test).
(TIF)

S5 Fig Analysis of PAR-6 levels in various embryonic extracts. Western blot analyses of embryonic extracts from animal of the specified genotype grown at 20°C and revealed with anti-PAR-6 and anti-alpha-tubulin antibodies. For each genotype, three extracts were prepared independently (top, middle, bottom) and each extract was probed in three separate western blot analyses (left, middle, right). The value under each lane corresponds to the ratio of PAR-6 over alpha-tubulin intensity normalized to tubulin levels in wild-type extracts. A different, longer exposure of the western blot shown in the top left was used for Fig. 5.
(TIF)
