# Supplementary figures and images for: CDK-1 and Two B-Type Cyclins Promote PAR-6 Stabilization during Polarization of the Early C. elegans Embryo
Source: PLoS One. 2015 Feb 6;10(2):e0117656. doi: 10.1371/journal.pone.0117656 (PMC4319824; doi:10.1371/journal.pone.0117656)

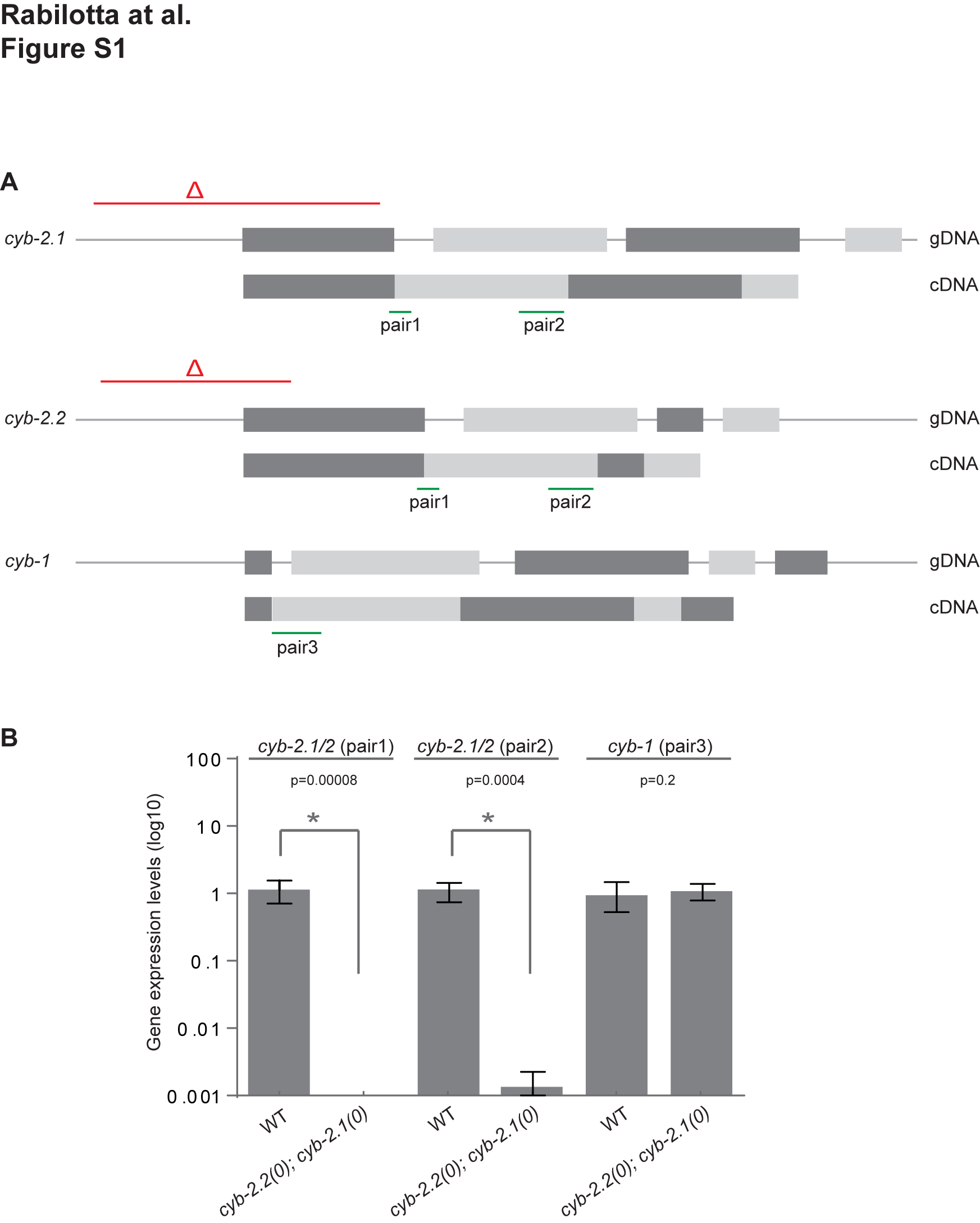

Supplement: S1 Fig — A) Schematic organization of the cyb-2.1 (top), cyb-2.2 (middle) and cyb-1 (bottom) loci and exon cDNA produced from each transcribed mRNA. Exons are depicted as light or dark grey boxes and non-coding regions (promoters, introns, flanking sequences) as lines. Position of deletions for the cyb-2.1(tm2027) and cyb-2.2(tm1969) alleles are depicted in red and the regions amplified by qPCR with each primer pair are shown in green. B) Relative gene expression ratios averaged from three biological samples for each strain. Bars indicate the 95% confidence interval of the mean (non-overlapping intervals denote significant differences at the 0.05 level). In each strain, expression levels of cyclin genes were normalized to the mean expression of cdc-42 and pmp-3. (TIF) [file pone.0117656.s001.tif]

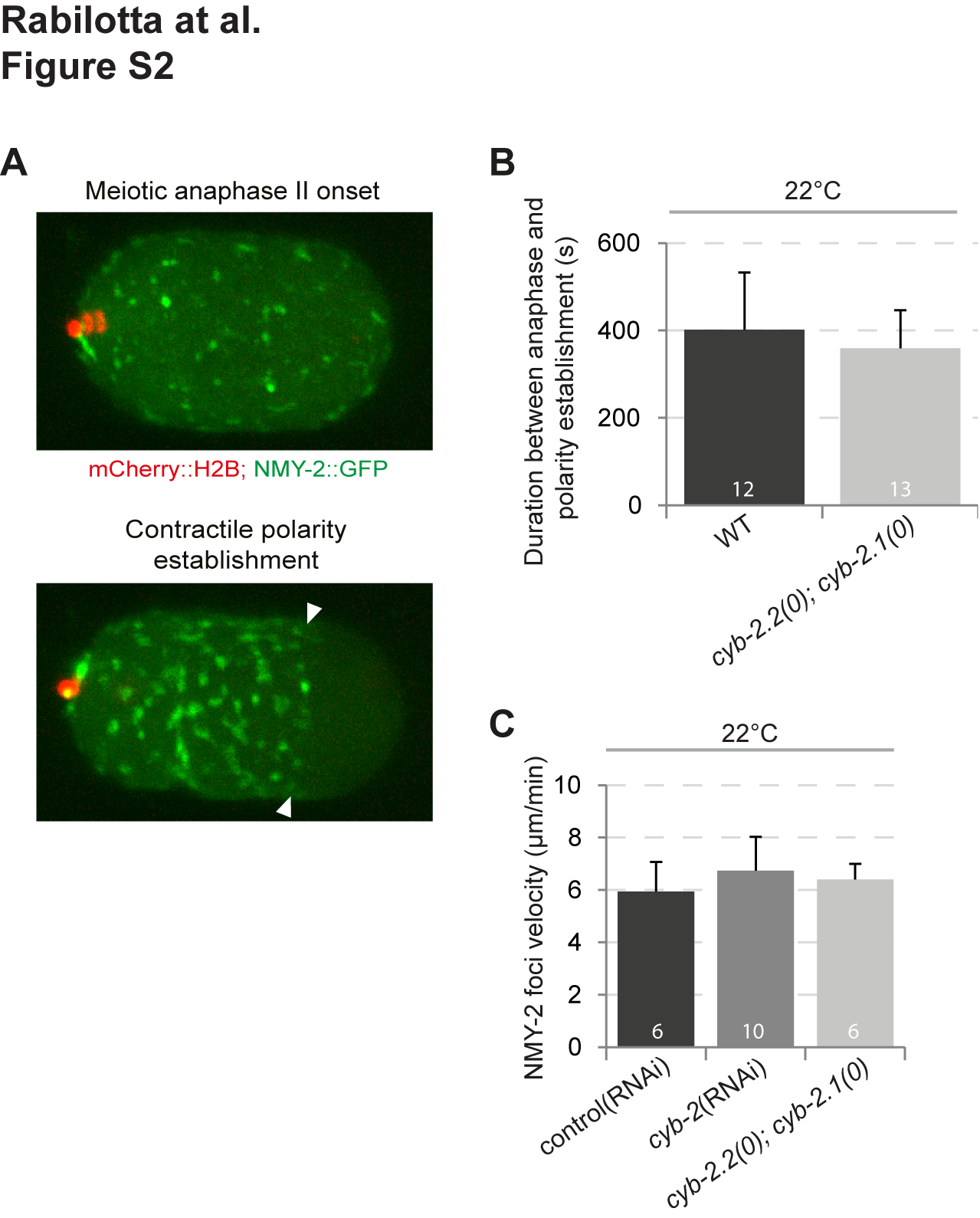

Supplement: S2 Fig — (A) Fluorescence images from time-lapse movies of control embryos undergoing meiosis II anaphase (left) and polarity establishment (right) and expressing NMY-2::GFP (green) and mCherry::H2B (red). White arrowheads point to the cortical domain boundary that is devoid of NMY-2::GFP. (B-C) Graphs reporting the time between meiotic II anaphase onset and contractile polarity establishment (B) and the velocity of NMY-2::GFP foci (C) in embryos of the specified genotypes grown at 22°C. Error bars represent standard deviation over the specified number of events (n). Values were not statistically different from control animals (p>0.05, Student’s t-test). (TIF) [file pone.0117656.s002.tif]

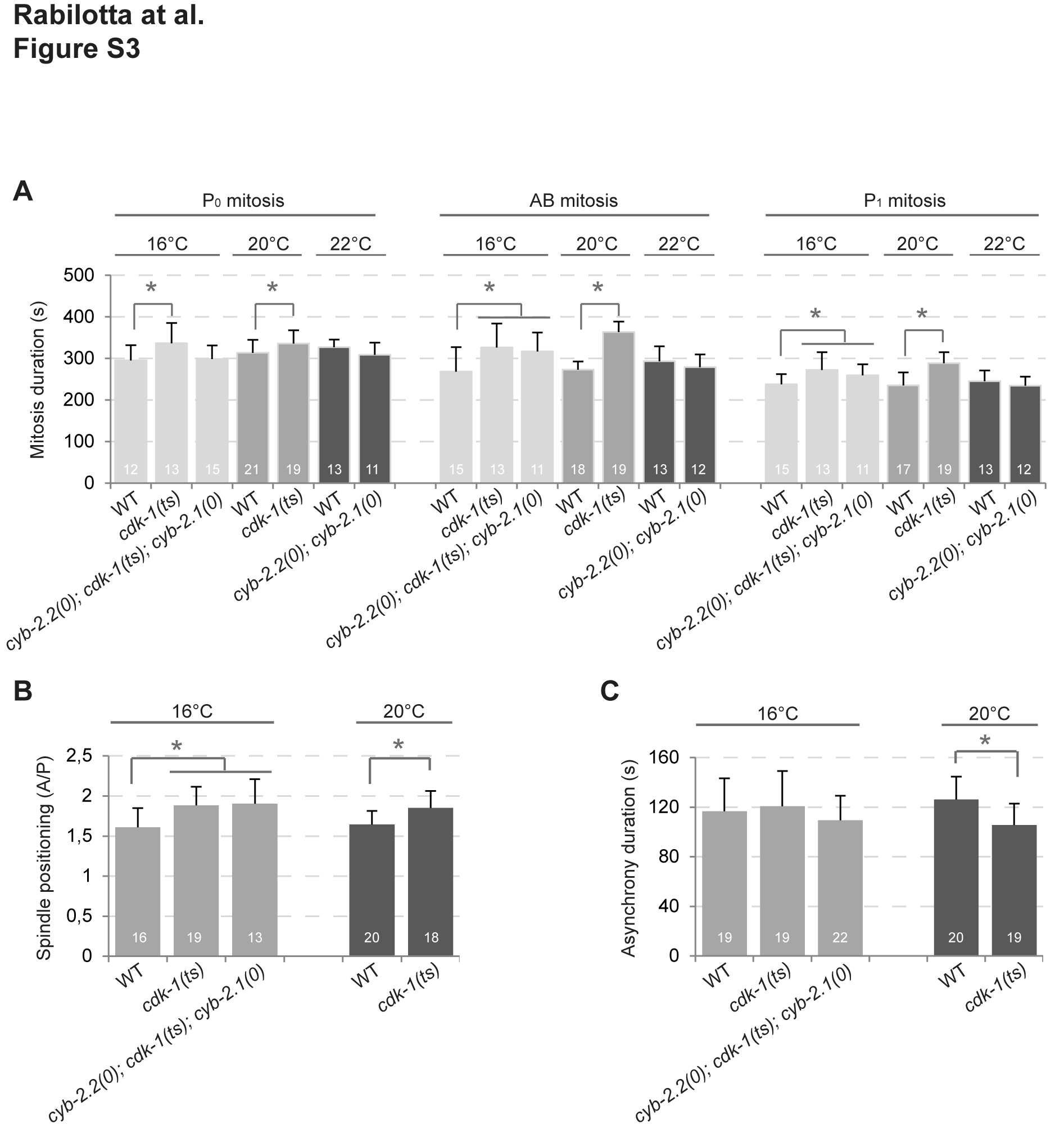

Supplement: S3 Fig — (A-C) Graphs reporting the duration of mitosis during the first two embryonic divisions (A), the measurements in 1-cell embryos of spindle positioning (B) and duration of asynchrony (C) in animals of the specified genotypes grown at the specified temperature. Error bars represent the standard deviation over the specified number of events (n). In all panels, asterisks indicate statistical significance with control animals (p≤0.05, Student’s t-test). (TIF) [file pone.0117656.s003.tif]

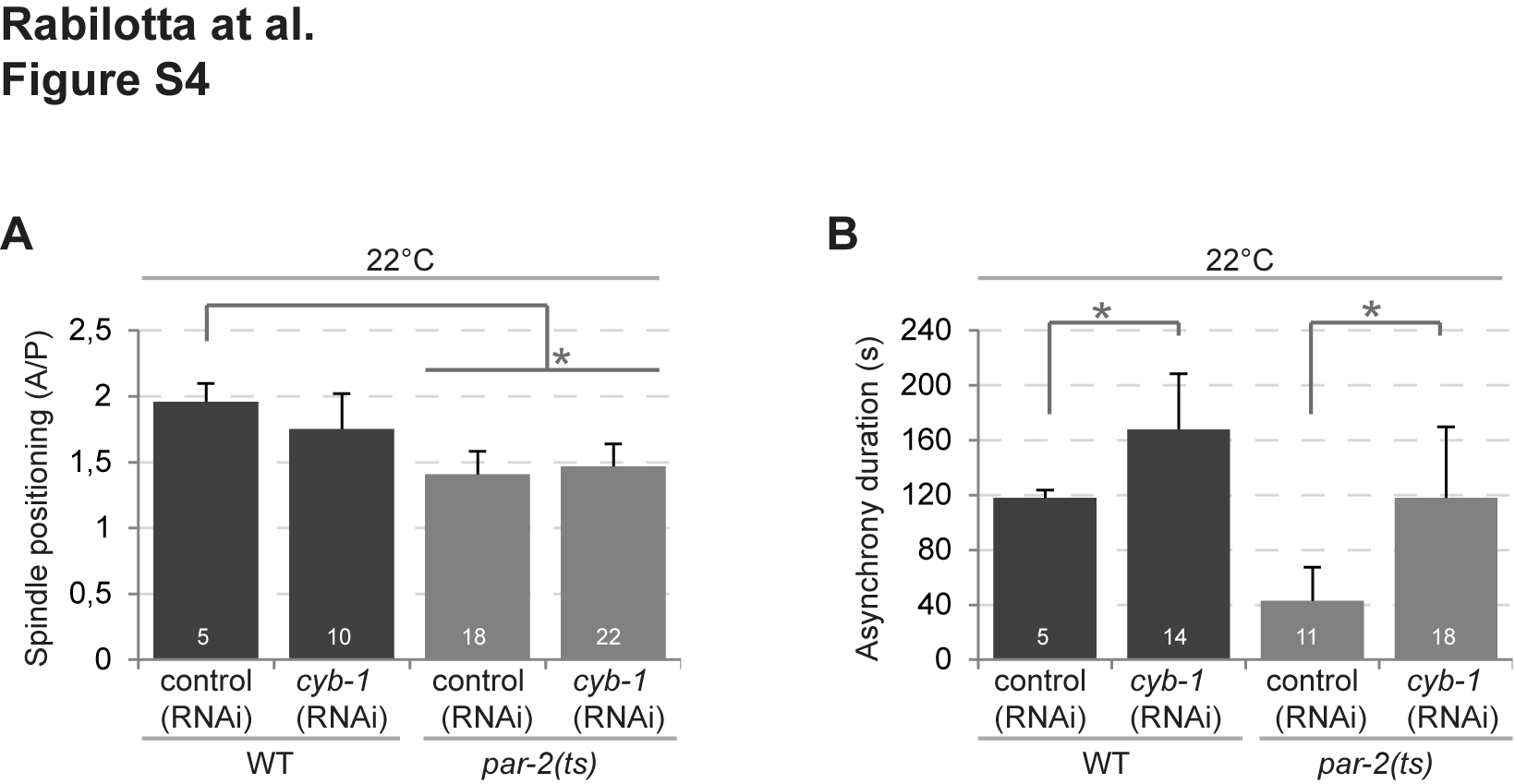

Supplement: S4 Fig — (A-B) Graphs reporting the measurements of spindle positioning (A) and duration of asynchrony (B) in 1-cell embryos of the specified genotypes grown at 22°C. Error bars represent the standard deviation over the specified number of events (n). In all panels, asterisks indicate statistical significance with control animals (p≤0.05, Student’s t-test). (TIF) [file pone.0117656.s004.tif]

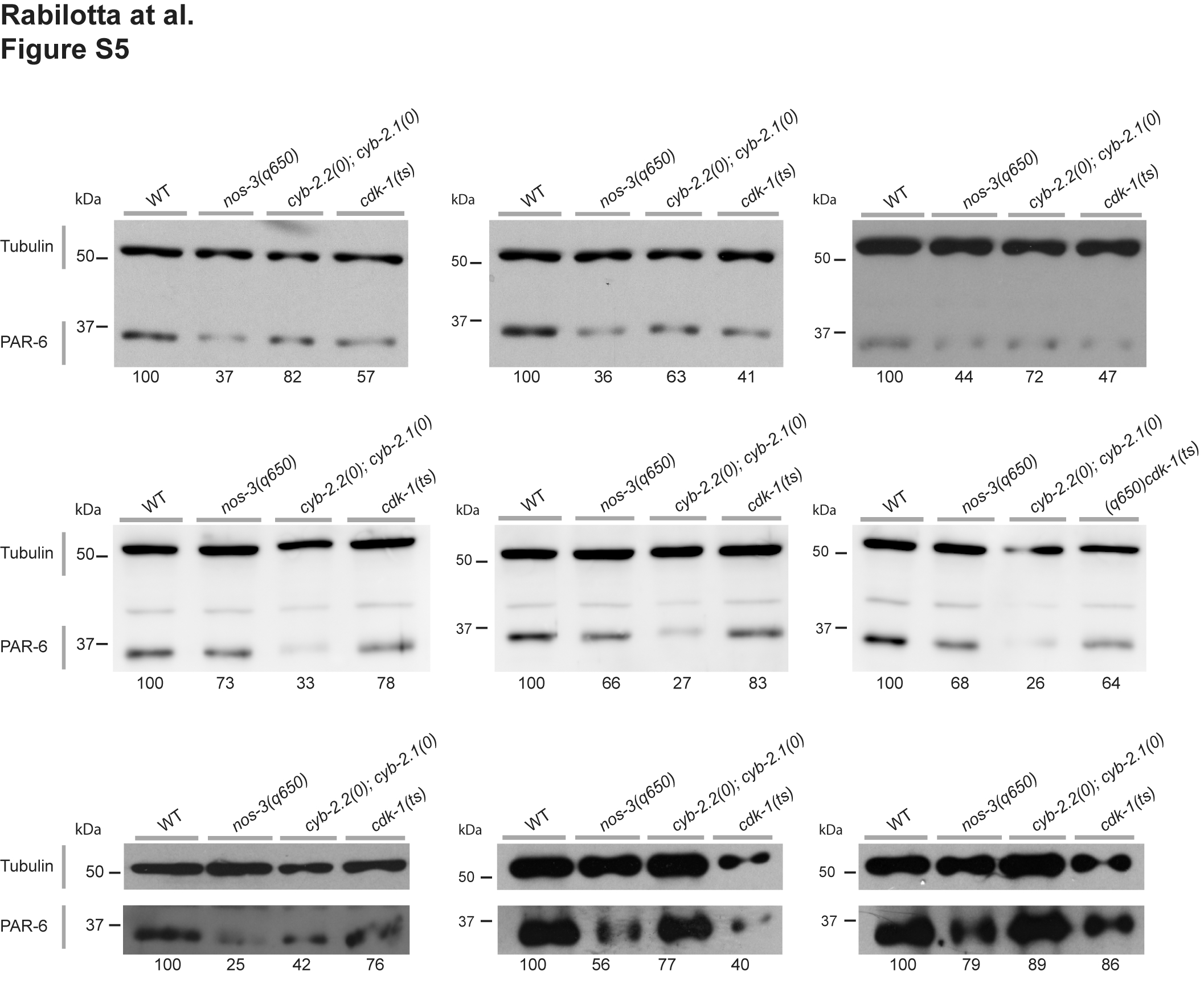

Supplement: S5 Fig — Western blot analyses of embryonic extracts from animal of the specified genotype grown at 20°C and revealed with anti-PAR-6 and anti-alpha-tubulin antibodies. For each genotype, three extracts were prepared independently (top, middle, bottom) and each extract was probed in three separate western blot analyses (left, middle, right). The value under each lane corresponds to the ratio of PAR-6 over alpha-tubulin intensity normalized to tubulin levels in wild-type extracts. A different, longer exposure of the western blot shown in the top left was used for Fig. 5. (TIF) [file pone.0117656.s005.tif]
